# Construction and validation of a U-type finite element model of an osteoporotic vertebral compression fracture

**DOI:** 10.3389/fbioe.2025.1617208

**Published:** 2025-09-17

**Authors:** Pengfei Li, Jihao Mu, Zhao Wang, Xiaochong Zhang, Yingze Zhang, Dengxiang Liu, Ao Li

**Affiliations:** ^1^ Postdoctoral Workstation, Xingtai City People’s Hospital, Xingtai, Hebei, China; ^2^ Postdoctoral Mobile Station, Hebei Medical University, Shijiazhuang, Hebei, China; ^3^ Department of Orthopedics, Harrison International Peace Hospital, Hengshui, Hebei, China; ^4^ Department of Research and Education, Xingtai City People’s Hospital, Xingtai, Hebei, China; ^5^ NHC Key Laboratory of Intelligent Orthopaedic Equipment, Department of Orthopaedics, Orthopaedic Research Institution of Hebei Province, The Third Hospital of Hebei Medical University, Shijiazhuang, Hebei, China; ^6^ Key Laboratory of Portal Hypertension and Cirrhosis of Hebei Provincial, Xingtai City People’s Hospital, Xingtai, Hebei, China

**Keywords:** osteoporotic vertebral compression fracture, U-type model, biomechanics, finite-element analysis, validation

## Abstract

**Background:**

An osteoporotic vertebral compression fracture (OVCF) is recognized as a common complication of osteoporosis. Biomechanical alterations in the affected and adjacent vertebrae have a significant influence on patient symptoms, treatment strategies, and clinical outcomes. Nevertheless, establishing an accurate model of OVCF remains a highly challenging task. In this study, a novel finite-element model of OVCF was developed and validated, and a comprehensive biomechanical analysis was conducted.

**Methods:**

Computed tomography data of the thoracolumbar spine (T12–L2) were collected from an OVCF patient and a healthy volunteer to establish the OVCF and normal models, respectively. Based on the normal model, U-type, V-type, and double-V-type finite element models were constructed. Intervertebral disks and articular cartilage were generated through a combination of appropriate materials and assemblies, followed by the development of three-dimensional finite-element biomechanical models. The magnitude and distribution of stress and displacement in these three models were evaluated and compared with those of the OVCF model under various directions of motion.

**Results:**

In the force distribution contour diagrams, the U-type model at the T12 vertebra most closely resembled the OVCF model, particularly in the directions of forward flexion, backward extension, left lateral bending, and left rotation. Force distribution patterns and stress concentration areas in all six directions were generally consistent between the U-type and OVCF models. At the L2 vertebra, the U-type model demonstrated the greatest similarity to the OVCF model in the direction of left lateral bending. At the T12/L1 intervertebral disk, no significant differences in the force distribution were observed among the four models. At the L1/2 intervertebral disk, the U-type and OVCF models showed the closest correspondence in the direction of forward flexion. In the displacement contour diagrams, the maximum displacements of the U-type model were found to be 1.7876 mm (forward flexion), 6.1564 mm (posterior extension), 4.6520 mm (left lateral bending), 6.2224 mm (right lateral bending), 3.4119 mm (left rotation), and 3.1601 mm (right rotation). Notably, in the direction of left lateral bending, the U-type model most closely approximated the displacement distribution of the OVCF model.

**Conclusion:**

The U-type finite-element model more accurately reproduces the biomechanical characteristics of OVCF and demonstrates high applicability.

## 1 Introduction

Osteoporosis (OP) is a systemic metabolic disorder primarily characterized by reduced bone mass and an increased risk of fragility fractures (Dai et al., 2022; Yu and Wang, 2022). Among these, osteoporotic vertebral compression fracture (OVCF) is the most common fracture in osteoporotic patients, occurring predominantly in individuals over the age of 50 (Huang et al., 2023; Li et al., 2024; Alimy et al., 2024). As the population ages, the incidence of OVCF continues to increase, leading to a growing number of patients suffering from acute or chronic pain, spinal deformities, and associated functional impairments (Li J. et al., 2023; Meng et al., 2025). These conditions substantially reduce the quality of life in the elderly population while increasing the socioeconomic burden on families and healthcare systems (Meng et al., 2025). Current treatment options for OVCF include conservative management, minimally invasive procedures, and open surgical interventions. Surgical approach selection—such as anterior, posterior, combined anterior–posterior, or posterior osteotomy—is typically based on the extent of vertebral collapse and deformity severity (Li J. et al., 2023). Dual-energy X-ray absorptiometry (DXA) remains the gold standard for osteoporosis diagnosis (Miranda et al., 2022; Li C. et al., 2023), while X-rays and magnetic resonance imaging (MRI) are employed for fracture detection and further assessment (Alsoof et al., 2022). However, the complexity of spinal anatomy and loading conditions poses significant challenges for traditional experimental biomechanics. Finite-element analysis (FEA), a computational technique that simulates the mechanical behavior of complex structures, has gained widespread application in vertebral fracture modeling due to advancements in computational power and modeling techniques (Guitteny et al., 2024). Despite its growing use, FEA-based modeling of vertebral compression fractures remains constrained by methodological limitations. Two commonly employed approaches—the V-type excision method and the double-V-type resection method—have been frequently referenced in the literature. Although the V-type model has been widely adopted, few studies have systematically described its construction procedures. Although this approach can simulate certain compression patterns, it fails to replicate the stress distribution associated with vertebral wedge deformities. The double-V-type approach, which involves creating angulated resections on the cranial and caudal vertebral surfaces, offers improved representation but still lacks clinical fidelity (Nakashima et al., 2018). To address these limitations, a novel morphology-based modeling approach has been proposed. This method seeks to enhance the simulation accuracy by incorporating anatomical and mechanical features that closely align with clinical fracture presentations. The proposed model aims to provide a more robust theoretical foundation for biomechanical research and offer precise guidance for the optimization of clinical treatment strategies.

## 2 Materials and methods

### 2.1 Case information

Through the hospital’s electronic medical record system, one OVCF patient with an L1 vertebral injury and a healthy volunteer without a history of spinal disease were selected as research participants. All participants underwent computed tomography (CT) scans of their entire lumbar spine for diagnostic or differential diagnostic purposes. The OVCF patient was 69 years old, with a height of 155 cm, a body mass of 55 kg, and a body mass index (BMI) of 22.89 kg/m^2^. The patient had no history of medical or surgical disease, physical disability, or trauma. A T-score of −3.5 and T < −2.5 meet the World Health Organization (WHO) diagnostic criteria for osteoporosis. The healthy volunteer was 15 years old, with a height of 180 cm and a body mass of 100 kg. This study was approved by the Ethics Committee of Harrison International Peace Hospital (Approval No. 2024246–1), and written informed consent was obtained from all participants.

### 2.2 Laboratory equipment and modeling, and analysis software

In this study, biomechanical mechanisms were investigated in depth through digital computer-aided modeling. Bone mineral density was measured and analyzed using a dual-energy X-ray absorptiometry (DXA) scanner (OsteoSys EXA-3000, South Korea, OsteoSys Co., Ltd.) at our hospital. A 64-slice high-resolution spiral CT scanner (Siemens Biograph mCT PET/CT, Germany, Siemens AG) was used to scan all segments of the thoracolumbar region, with a slice thickness of 0.625 mm, a tube voltage of 140 kV, and a current of 200 mA. A total of 507 images in sagittal, coronal, and axial planes were obtained. The CT data were converted to DICOM format and stored on a CD-ROM. The acquired two-dimensional CT images were then imported into Mimics 21.0 modeling software for the extraction and transformation of the original model contours. Geomagic 2017 software was used for feature modification, surface smoothing, surface fitting, and separation of cortical and cancellous bones. SolidWorks 2024 was utilized for structural generation and assembly, while Ansys Workbench 2022 R^1^ was employed for assigning material properties, mechanical meshing, ligament construction, and biomechanical analysis.

### 2.3 Construction of the finite-element model of the normal T12–L2 thoracolumbar spine

The DICOM-format CT data from the healthy volunteer were extracted and imported into Mimics 21.0 software to establish the finite-element model of the normal T12–L2 thoracolumbar spine. The “Bone Window Rendering Thresholding” function was applied to segment the imported files at the bone tissue–soft tissue interface, with a threshold range set from 259 to 1906 HU. The thoracolumbar spine model was reconstructed layer by layer using the “Edit Masks” tool, allowing for precise segmentation of the vertebrae and other structural units. The final model was saved in STL format. Structural unit optimization—including smoothing, grinding, denoising, surface reconstruction, and solidification—was performed using Geomagic Warp 2017 software. Simulated intervertebral disks, endplates, and cartilage were generated in SolidWorks 2024, with the intervertebral disks further subdivided into the nucleus pulposus and annulus fibrosus.

### 2.4 Material properties and contact properties

**FIGURE 1:**
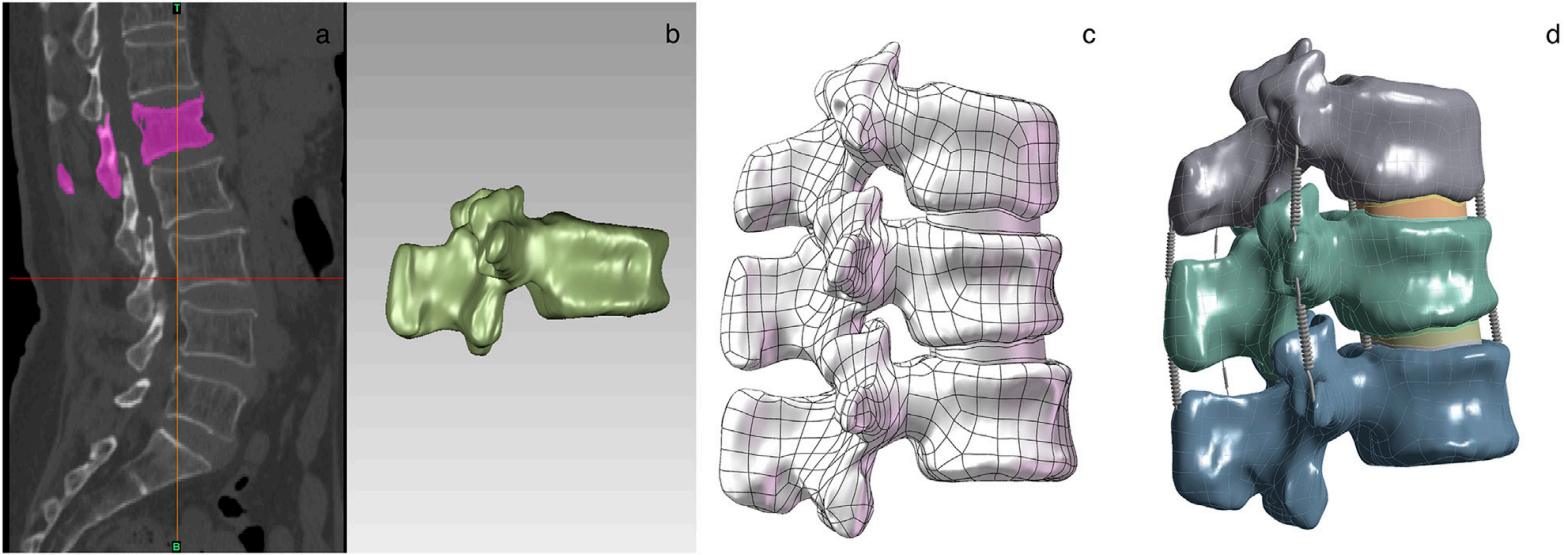
T12-L2 spine model construction process: **(a)** Model extraction and format conversion; **(b)** Smoothing processing and protrusion removal; **(c)** Structural optimization and assembly; **(d)** Parameter setting and physical solving.

The parameterization of the thoracolumbar segment model was done with the help of Ansys 2022 software. The material properties of the osteoporotic vertebrae were modeled by assuming that all structures were made of homogeneous linear elastic materials. The assignment of material properties to parts of the 3D model was based on those used in recent OVCF studies (Table 1) (Huang et al., 2023; Li et al., 2024; Pan et al., 2024; Yu et al., 2024) (Table 2) (Constant and Murley, 1987). Goel et al. (1993), Zhang et al. (2010), Huang et al. (2023) proposed that the modulus of elasticity of cortical bone, vertebral endplates, and posterior vertebral structures of the thoracolumbar segment of the spine was reduced by 33%, and the modulus of elasticity of the cancellous bone was reduced by 66%, and osteoporosis was simulated by decreasing the modulus of elasticity of each vertebral type by a certain amount. The model is meshed by controlling the mesh type and size to ensure that the computational accuracy meets the analysis requirements. The mesh size of the cartilage is set to 0.5 mm, and the mesh size of the rest of the model is set to 2 mm. The ligament portion is replaced with a spring set to stretch only (Che et al., 2022). The contact of articular cartilage with other surfaces was defined as frictional, with a friction coefficient of 0.1, and the contact of all other surfaces was set as bound (Figure 1).

**TABLE 1:** Material properties of each part of the model.

Spinal component	Young’s modulus (MPa)	Poisson’s ratio
Normal cortical bone	12,000	0.3
Osteoporotic cortical bone	8,040	0.3
Cancellous bone	132	0.2
Osteoporotic cancellous bone	34	0.2
Normal endplate	1,000	0.4
Osteoporotic endplate	670	0.4
Nucleus pulposus	1	0.49
Fiber ring	4.2	0.45
Anterior longitudinal ligament	20	0.3
Posterior longitudinal ligament	20	0.3
Intertransverse ligament	40	0.45
Interspinous ligament	12	0.45
Supraspinous ligament	12	0.45
Yellow ligament	20	0.45

**TABLE 2:** Material parameters of the model ligaments.

Part	K_1​_/(N⋅mm^−1^)	n	K_2_/(N⋅mm^−1^)
Anterior longitudinal ligament	43.70	2	8.74
Posterior longitudinal ligament	29.15	2	5.83
Intertransverse ligament	19.04	4	2.39
Interspinous ligament	0.95	2	0.19
Supraspinous ligament	76.90	2	15.38
Yellow ligament	47.25	2	15.75

### 2.5 Model construction of OVCF, V-type, double-V-type, and U-type

V-type Fracture Model: The V-type fracture model was constructed in Geomagic 2017 based on vertebral data from the healthy volunteer. Beginning at the posterior vertebral edge vertex, a diagonal penetrating line was created using through-mode line selection, extending anteriorly until 27.5% compression was achieved. The model was then divided into resected and retained portions. After removal of the resected section, the superior surface of the vertebra was reconstructed using the fill command. Feature removal and spike elimination functions were subsequently applied to prevent stress concentration, resulting in the final simulated V-type fracture model.

Double-V-type Fracture Model: The double-V-type fracture model was constructed in Geomagic 2017 using vertebrae from the healthy volunteer. Through-mode line selection was applied at identical angles to both the superior and inferior surfaces from the posterior edge vertex, extending anteriorly until 26.6% compression was achieved. Following segmentation and removal of the resected portions, the superior surface was reconstructed using the fill command. Feature removal and spike elimination were subsequently performed to minimize stress concentration, resulting in a simulated double-V-type fracture model.

U-type Fracture Model: The U-type fracture model was constructed in Geomagic 2017 using vertebrae from the healthy volunteer. The brush tool was uniformly applied across the superior and inferior surfaces, with iterative offset adjustments used to create an inward depression until 27.4% compression was achieved. A sculpting knife was then used to blunt sharp edges and prevent stress increasers, followed by the application of quick smoothing and sandpaper functions to remove excess material, resulting in the finalized U-type fracture model.

**FIGURE 2:**
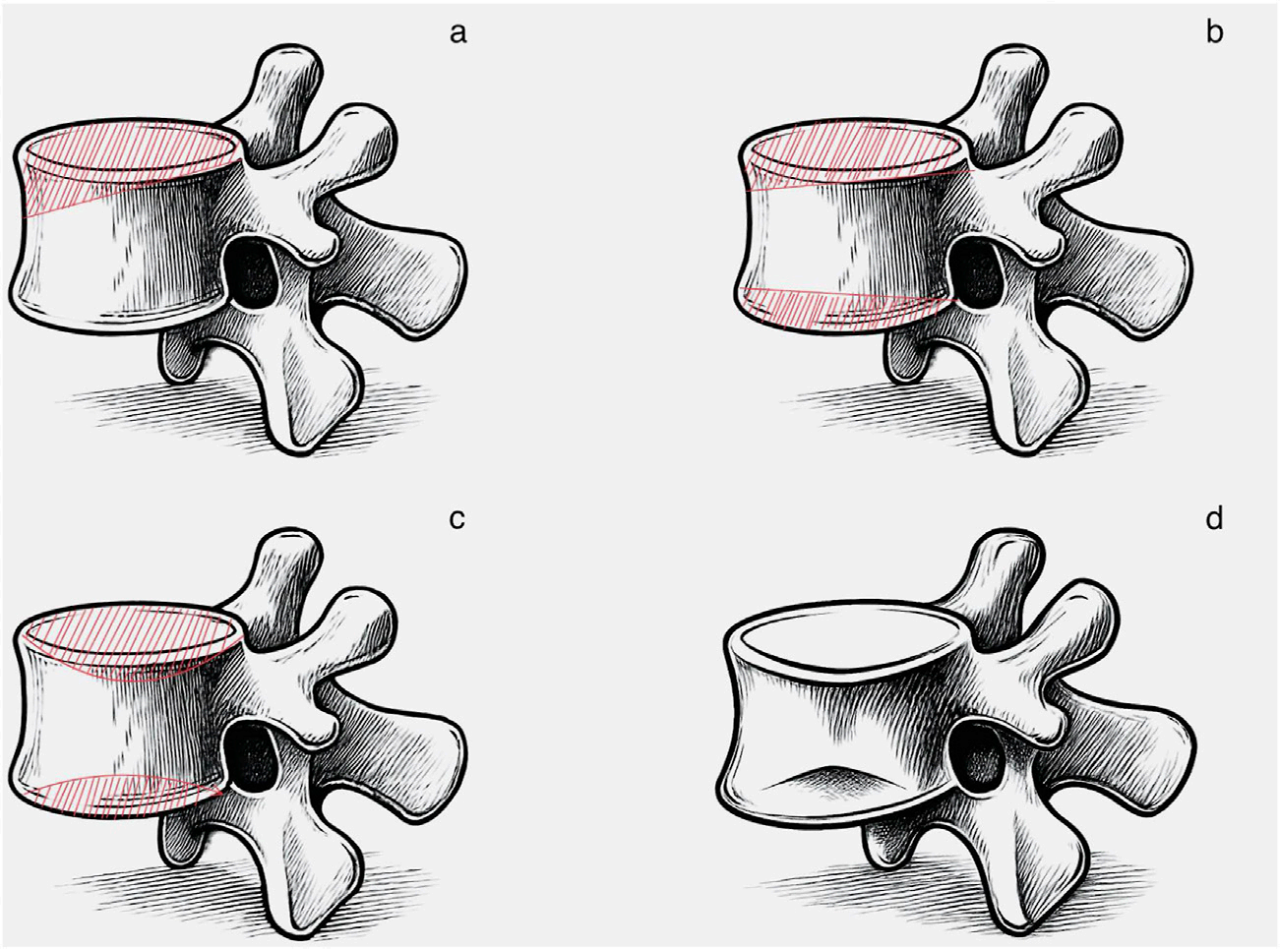
Construction of four finite-element models: **(a)** V-type; **(b)** Double-V-type; **(c)** U-type; **(d)** Extractive.

Extracted Fracture Model: A biconcave fractured vertebra, confirmed through clinical assessment, was processed using Geomagic 2017. Sandpaper and feature removal tools were applied to achieve comprehensive surface blunting while preserving the native anatomical morphology. The model was then adjusted to 26.5% compression to generate the extracted fracture model (Figure 2).

Referring to the EVOSG typing system (Ismail et al., 1999), this patient’s fracture type was assessed morphologically as biconcave based on basic radiographic measurements, including the degree of vertebral wedging (localized kyphosis) and vertebral height loss (expressed in millimeters or as a percentage), in compression fractures (Ruiz Santiago et al., 2016). Referring to the Genant semi-quantitative (SQ) typing system (Genant et al., 1993), the degree of compression of the fractured vertebral body of the OVCF model was 26.5% and the degrees of compression of the other three models were adjusted to be close to that of the OVCF model. Consequently, a comparative analysis was conducted between moderate biconcave vertebral fractures and other morphologically distinct yet compression-matched moderate vertebral fractures. By maintaining identical compression ratios with varying fracture morphology, the variables were effectively isolated to ensure more targeted and reliable research outcomes. This is consistent with the fact that a reduction in vertebral height of >15% is the morphometric criterion required for an imaging diagnosis of a new vertebral fracture (Ruiz Santiago et al., 2016). The degree of vertebral body compression is obtained by taking the posterior margin of the vertebral body as the pre-compression and the bottom end of the compression as the post-compression. That is, the degree of vertebral body compression = [(height of the posterior margin of the vertebral body - height of the lowest end of the vertebral body) ÷ height of the posterior margin of the vertebral body] × 100%.

### 2.6 Control mode and boundary condition

Future computational models should incorporate paraspinal musculature and intra-abdominal pressure to enhance the clinical relevance of vertebral fracture simulations, given their influence. In the current model, all nodes on the inferior surface of the L2 vertebra were constrained in the X, Y, and Z directions. The coordinate system of the thoracolumbar spine segment was used as a reference frame to assign mechanical parameters corresponding to different degrees of freedom. Loading conditions were then defined based on the X-, Y-, and Z-axes to simulate various motion states. A compressive load of 500 N was applied coaxially along the Z-axis to the coupling node on the superior surface of the T12 vertebral body, simulating the vertical loading experienced by the thoracolumbar spine in an upright posture (Pan et al., 2024). This load was uniformly distributed across the entire surface. To simulate flexion and extension, torques of +10 N/m and −10 N/m were applied about the Y-axis. Side-bending motions were modeled by applying torques of +10 N/m and −10 N/m about the X-axis. For rotational loading, torques of +10 N/m (left rotation) and −10 N/m (right rotation) were applied about the Z-axis. These boundary conditions were used to solve for the equivalent forces and displacements of the vertebral bodies under different physiological motion scenarios.

### 2.7 Statistical methods

All data were analyzed using SPSS Statistics version 27.0. For continuous variables, normality and homogeneity of variance were first assessed based on the maximum displacement values across the four fracture models. When both assumptions were met, one-way analysis of variance (ANOVA) was performed, followed by *post hoc* comparisons using the Student–Newman–Keuls q (SNK-q) test. For data that did not satisfy normality assumptions, the nonparametric Wilcoxon rank-sum test (Mann–Whitney U test) was applied. Results are reported as the mean ± standard deviation, with statistical significance set at P < 0.05.

## 3 Results

### 3.1 Validation of the model

The current finite-element model was validated under combined loading conditions of pure moments and follower loads. Normal bone density was first assigned to the spinal materials, and the model’s mobility was evaluated in all six anatomical directions. No significant differences were observed when compared with the experimental results reported in previous studies (Schmoelz et al., 2010; Huang et al., 2023). Subsequently, osteoporotic bone density was assigned to the model for force analysis.

### 3.2 Force analysis

**FIGURE 3:**
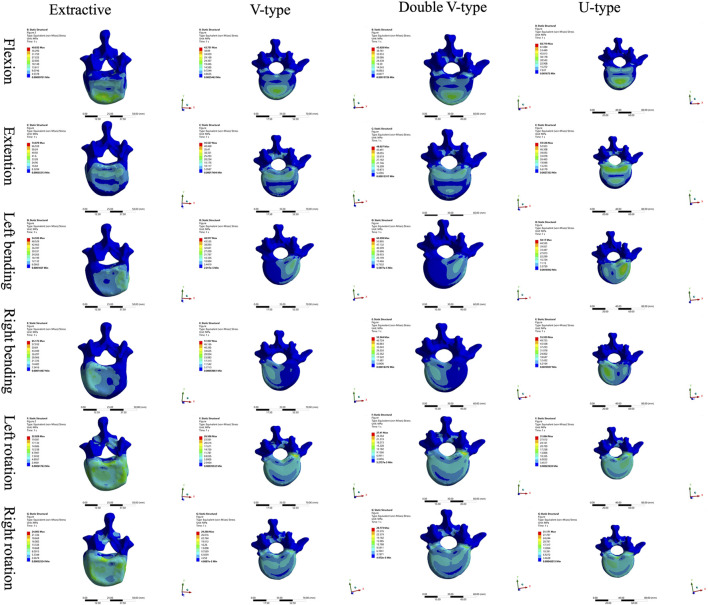
Cloud view of the T12 vertebral force distribution.

Comparison of T12 vertebral force clouds revealed significant variations in force distribution under different loading conditions (Figure 3). According to the stress distribution of the four fracture models in six directions, the maximum stress was primarily concentrated in the cortical bone, and from the stress distribution on the upper surface of the cortical bone, it could be seen that, in the forward flexion direction, the force of the four models was concentrated on the anterior edge of the vertebral body and then distributed along the posterior edge of the vertebral body, which was more obvious in the OVCF and U-type models; in the direction of the posterior extension, the forces of all four models were concentrated on the posterior margin of the vertebral body, with the OVCF model being the most obvious; in the direction of left lateral bending, the forces of all four models were concentrated on the left side of the vertebral body, with the OVCF and U-type models being the most obvious; in the direction of the right lateral curvature, the forces of all four models were concentrated on the right side of the vertebral body, and there was no significant difference among the four models; in the direction of left rotation, the OVCF and U-type models were distributed on the left side of the vertebral body, while the V-type and double-V-type models were distributed in the region where the vertebral body was connected to the pedicle; in the rightward direction, the force on the U-type vertebra was concentrated on the right edge of the vertebrae, and the distribution of the force on the other three types of vertebrae was not obvious.

**FIGURE 4:**
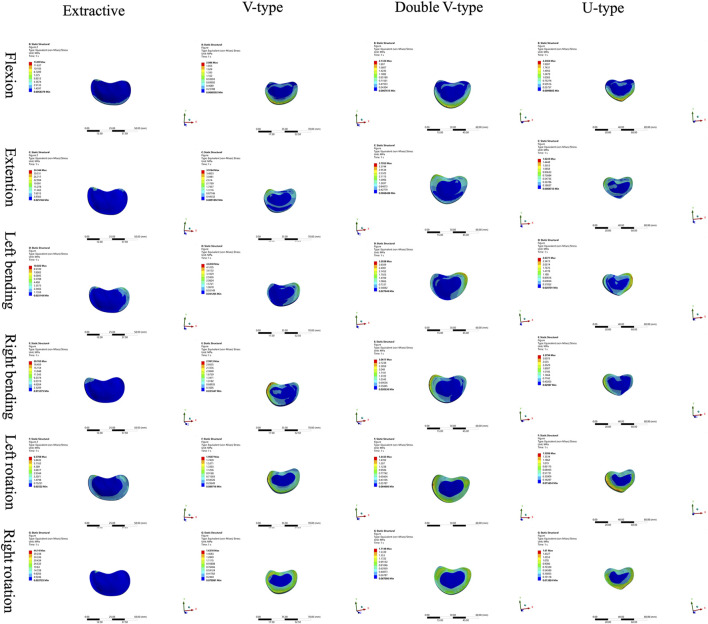
Cloud view of the force distribution on T12 and L1 intervertebral disks.

**FIGURE 5:**
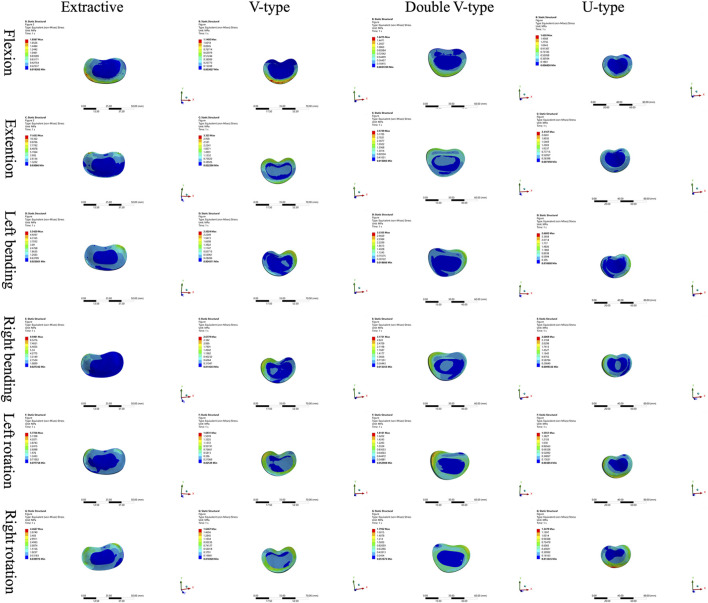
Cloud view of the force distribution on the L1 and L2 intervertebral disks.

Comparison of the four intervertebral disk force cloud models revealed significant variations in force distribution among the models under different loading conditions (Figures 4, 5). In the disks of T12 and L1, there was no significant difference in the distribution of force among the four models. In the disks of L1 and L2, the U-type model was the closest to the OVCF model in the direction of forward flexion. There was no significant difference in the patterns of stress distribution of the four models in any other direction.

**FIGURE 6:**
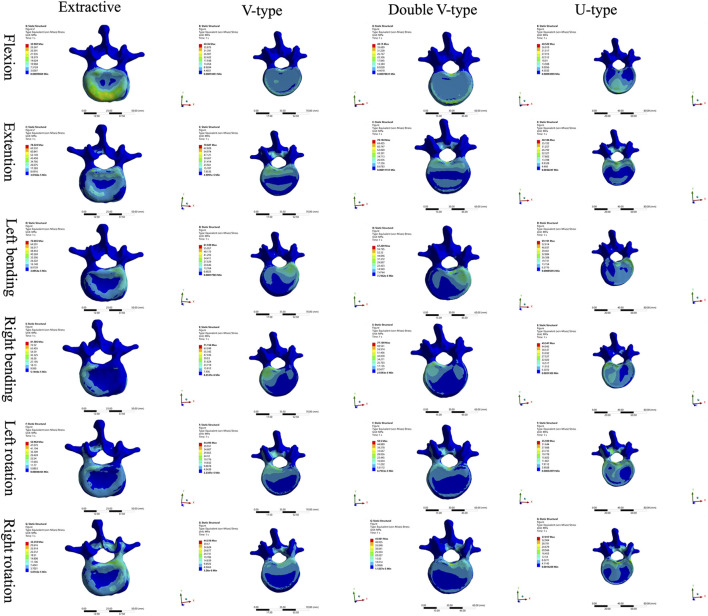
Cloud view of the L1 vertebral force distribution.

Comparison of L1 vertebral body stress clouds revealed significant differences in stress distribution under varying loading conditions (Figure 6). According to the stress distribution of the four fracture models in the six directions, the maximum stress was concentrated in the cortical bone, and the stress distribution on the upper surface of the cortical bone could show that, in the forward-flexion direction, the U-type model was closer to the OVCF model, which were all concentrated in the anterior margin of the vertebral body, and there was no specificity in the stress distribution of the V-type and double-V-type models; in the left-rotation direction, the double-V-type model was closer to the OVCF model, and it was distributed in the left rotation direction; the double-V-type model was closer to the OVCF model and was distributed on the right side of the posterior vertebral body edge; in the right rotation direction, the U-type model was closer to the OVCF model and was distributed on the left side of the posterior vertebral body edge; in the remaining directions, the four models were in good agreement.

**FIGURE 7:**
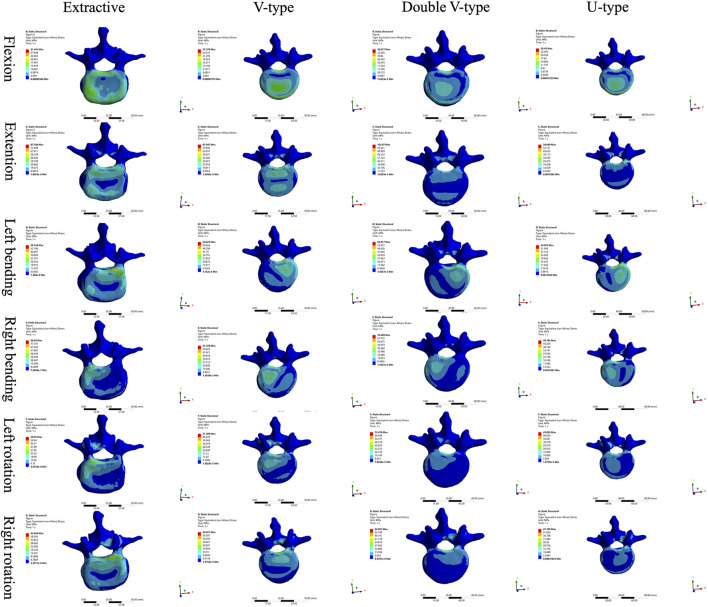
Cloud view of the L2 vertebral force distribution.

Comparison of L2 vertebral body stress clouds revealed significant differences in stress distribution under different loading conditions (Figure 7). According to the stress distribution of the four fracture models in six directions, the maximum stress was concentrated in the cortical bone, and from the stress distribution on the upper surface of the cortical bone, it could be seen that, in the direction of posterior extension, the V-type model was closer to the OVCF model, with a significant part concentrated on the posterior edge of the vertebral body and a small part in the anterior edge of the vertebral body; in the direction of the left lateral curvature, the U-type was closer to the OVCF model, and was concentrated in the posterior edge of the left side of the vertebral body; the remaining directions showed no significant difference.

**FIGURE 8:**
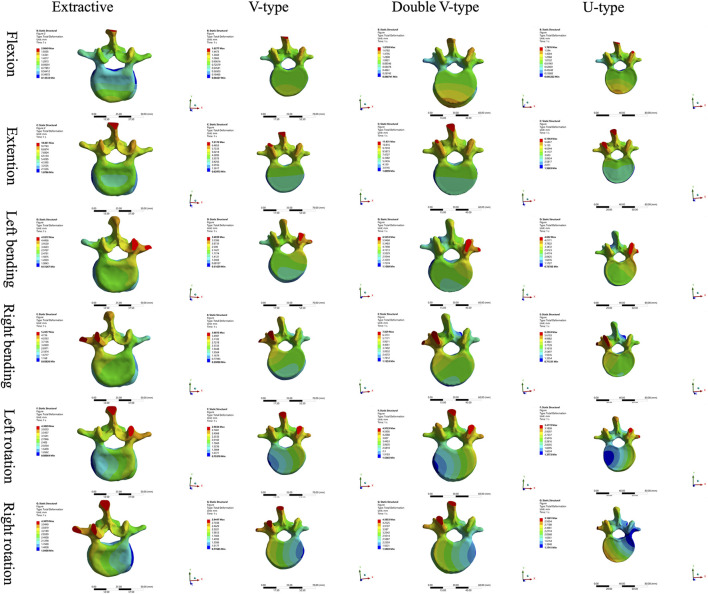
Cloud view of the L1 vertebral displacement distribution.

**FIGURE 9:**
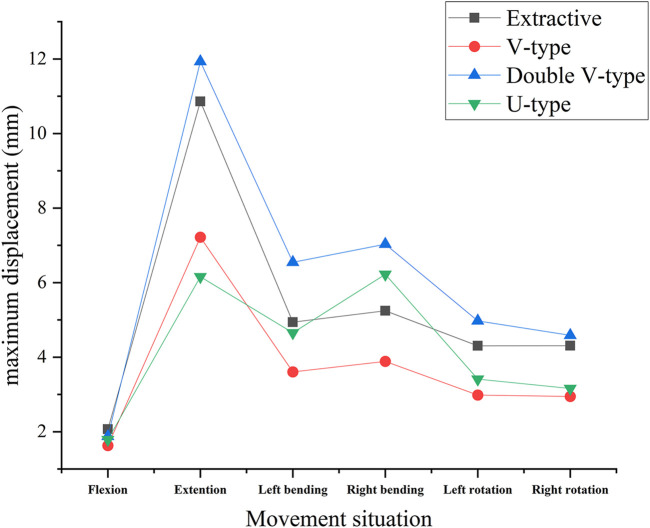
Variation of maximum displacement in different states of the four fracture models.

**FIGURE 10:**
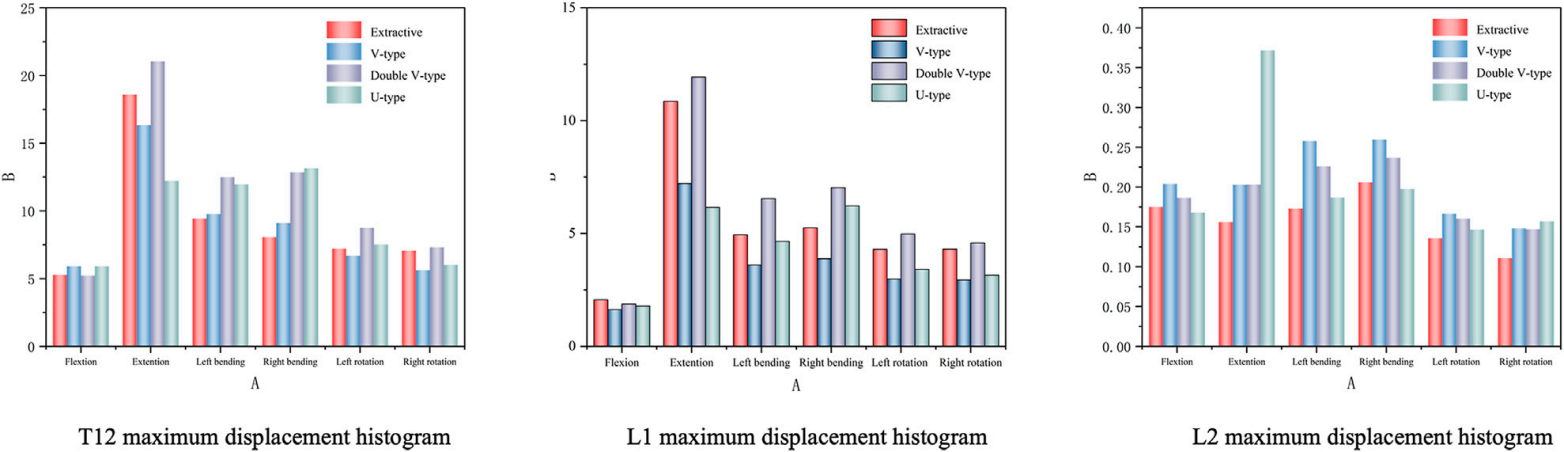
Histogram of the distribution of maximum displacement values for each vertebra.

**FIGURE 11:**
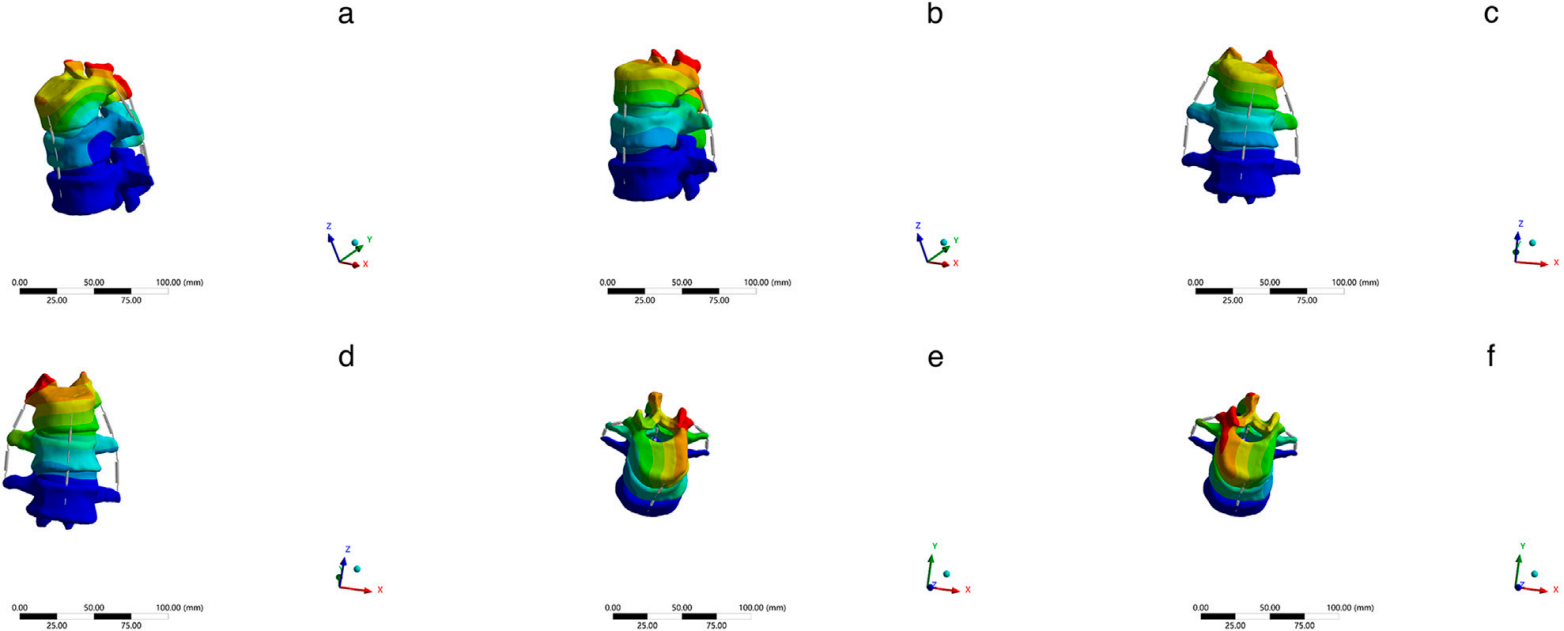
The displacement states of the extracted model in different loading directions. **(a)** Flexion **(b)** Extension **(c)** Left bending **(d)** Right bending **(e)** Left rotation **(f)** Right rotation.

A comparative analysis of maximum vertebral displacements across six loading directions (Table 3; Figures 8–10) revealed that the U-type fracture model exhibited displacements of 1.7876 mm (axial), 6.1564 mm (flexion), 4.6520 mm (left lateral bending), 6.2224 mm (right lateral bending), 3.4119 mm (left rotation), and 3.1601 mm (right rotation) at the L1 level. Morphological comparisons demonstrated the closest displacement matching between the U-type and the extracted models in left lateral bending, while the double-V-type and the extracted models showed maximal similarity during extension and right rotation. A statistical analysis confirmed no significant intergroup differences (p > 0.05). A multi-vertebral (T12-L2) assessment further identified the closest biomechanical agreement between the U-type and the extracted models in flexion, left lateral bending, and left rotation. Representative displacement patterns of the U-type model under multidirectional loading are illustrated in Figure 11.

**TABLE 3:** Maximum displacements in different states for the four fracture models (x ± s,mm).

Group	Extractive (n = 6)	V-type (n = 6)	Double V-type (n = 6)	U-type (n = 6)	F	P
T12 displacement value	9.27 ± 1.94	8.90 ± 1.64	11.28 ± 2.30	9.46 ± 1.36	0.33	0.80
L1 displacement value	5.29 ± 1.20	3.71 ± 0.77	6.16 ± 1.37	4.23 ± 0.72	1.07	0.38
L2 displacement value	0.15 ± 0.14	0.21 ± 0.19	0.19 ± 0.15	0.20 ± 0.34	0.99	0.42

“n” represents the models in different loading directions.

## 4 Discussion

The force distribution and maximum displacement observed in the finite-element analysis served as key indicators for predicting the risk of spinal re-fracture. The force distribution of the T12 vertebra was examined first. In the directions of forward flexion, backward extension, left lateral bending, and left rotation, the U-type model exhibited force patterns most similar to those of the OVCF model, whereas no significant similarities were observed in the other directions. Analysis of the force and displacement distributions on the superior surface of the L1 vertebra revealed that the U-type model preserves consistent stress patterns across all six motion directions when compared to the extracted model. The areas of stress concentration were approximately equivalent, and the displacement patterns exhibited strong spatial alignment with the stress distributions. Regions showing the most significant displacement changes corresponded closely with high-stress zones, which may improve the predictive accuracy for potential damage. However, no statistically significant differences in force or displacement distributions were observed among the four models. In the L2 vertebra, force analysis under posterior extension showed that the V-type model more closely resembled the OVCF model, with stress primarily concentrated on the posterior margin and partially on the anterior margin. Under left lateral bending, the U-type model showed the greatest similarity to the OVCF model. Finally, the intervertebral disks were assessed. In the T12/L1 disk, no substantial differences in the force distribution were observed among the four models. In the L1/L2 disk, the U-type model demonstrated the greatest similarity to the OVCF model in the forward flexion direction, while no significant differences were noted in other directions.

The analysis results revealed that, among all models, the U-type model most closely resembled the OVCF model in terms of force distribution patterns in the T12, L1, and L2 vertebral bodies, along with the intervertebral disks, and the maximum displacement of the L1 vertebra. This suggests that the fracture modeling approach used in the U-type model is more suitable for replicating wedge-like vertebral deformities. In contrast, the V-type model demonstrated substantial differences in stress distribution compared to the OVCF model, likely due to morphological dissimilarities.

Each simulation model appeared suited to a specific type of vertebral compression. The V-type model, for instance, was more appropriate for simulating anterior compression. However, because vertebral compression is often bidirectional, removing bone solely from the superior edge fails to adequately represent real-world anterior compressive deformities. To address this limitation, a double-V-type model was proposed. This approach allowed for the controlled removal of bone from both superior and inferior margins, producing a more accurate representation of anterior compression consistent with clinical presentations. Nevertheless, in the current experiment, the posterior compression component in the double-V-type model remained limited. As such, posterior compression was not explicitly analyzed. A posterior compression model may be constructed by initiating bone removal from the anterior margin and extending it toward the posterior aspect of the vertebral body. Considering the diversity of clinical cases, an ideal, controllable fracture model may be achieved by adjusting the initial cutting height in anterior–posterior compression models or by modifying the anterior and posterior edge heights in wedge-type compression models through surface polishing techniques.

According to the European Osteoporosis Spine Study Group typology, vertebral fractures are classified as normal, extruded, biconcave, or wedge, depending on the location of the deformity within the vertebral body (Ismail et al., 1999). The most commonly employed fracture modeling techniques—V-excision and double V-excision—are primarily suited for simulating wedge-type deformities. However, findings from the present study indicate that these approaches were not appropriate for modeling biconcave spinal compression fractures, highlighting the need for a novel resection strategy. Under idealized conditions, a concave arc surface exerts a supporting force that consistently points toward the center of curvature and dynamically adjusts in response to changes in the object’s position. When an object remains stationary or experiences only minor displacements on such a surface, the components of the supporting and gravitational forces more readily achieve equilibrium. This promotes positional stability and reduces the likelihood of deviation from a balanced state. In contrast, the inclined plane generated by the V-type osteotomy fails to provide posterior-directed support to the superior vertebral body. This leads to increased risk of intervertebral misalignment and limits the model’s capacity to replicate the biomechanical behavior characteristic of wedge deformities. The U-type construction method addressed this limitation by more accurately simulating the mechanical characteristics of biconcave spinal compression fractures. It enabled the prediction of potential fracture sites and key stress-loading regions by analyzing the stress distribution and identifying zones of maximum stress. The development of this novel fracture model offers a more faithful simulation of OVCF and provides a theoretical foundation for guiding clinical treatment strategies.

In this study, fracture models constructed using different techniques were initially compared with real clinical cases. The comparison results indicate that the biconcave construction method most closely resembled the extracted cases in terms of stress magnitude, distribution, and displacement. However, due to morphological variability among fracture types, certain cases may exhibit greater similarity to other modeling approaches with respect to biomechanical characteristics. According to the three-column theory of the spine, approximately 85% of spinal load is transmitted through the anterior and middle columns, while the posterior column bears the remaining 15% (Zhang et al., 2022; Denis, 1983). This framework suggests that spinal instability may increase the risk of vertebral recompression. Therefore, future studies should incorporate dynamic musculoskeletal simulations to improve the physiological realism and clinical relevance of spinal finite-element models (Fan et al., 2022).

In this study, restricting the loading conditions to uniaxial vertical compression overlooked the influence of physiologically relevant multidirectional stresses, such as shear and torsion. To reduce the discrepancy between experimental simulations and clinical reality, all models were constrained at the inferior surface of the L2 vertebral body. Future studies should incorporate theoretical analyses of complex loading patterns and assess the biomechanical implications of multidirectional forces for clinical applications (Huang et al., 2023). In osteoporotic models, the spatial distribution of bone mineral density (BMD) is frequently simplified by assuming homogeneous material properties. This approach reduces the computational complexity and facilitates early-stage model development. While uniform elastic parameters may provide preliminary insights, they fail to account for regional variations in BMD and trabecular architecture. In the present study, 67% of normal BMD was adopted as the baseline value; however, the use of a single uniform value was methodologically inadequate given the heterogeneity of osteoporotic bone (Pan et al., 2024). Therefore, more advanced modeling strategies are warranted. Post-fracture physiological changes introduced additional complexity. The material properties of lumbar vertebrae are dynamically altered due to bone loss at the fracture site, vascular disruption, and changes in the local inflammatory microenvironment. These factors result in transient reductions in BMD and elastic modulus. A key limitation of the present study was the absence of time-dependent analysis, particularly in relation to bone remodeling processes. This omission restricted the clinical translatability of the findings. To more accurately predict postoperative recovery trajectories, future research must incorporate these dynamic biological processes.

## 5 Conclusion

The U-type finite-element model demonstrates high applicability in simulating osteoporotic vertebral compression fractures. This modeling approach enables more accurate reproduction of the biomechanical characteristics of vertebral fractures, thereby providing a robust theoretical foundation for clinical management and a scientific basis for optimizing relevant treatment strategies.

## Data Availability

The raw data supporting the conclusions of this article will be made available by the authors, without undue reservation.
